# Factors affecting commencement and cessation of betel quid chewing behaviour in Malaysian adults

**DOI:** 10.1186/1471-2458-11-82

**Published:** 2011-02-07

**Authors:** Wan MN Ghani, Ishak A Razak, Yi-Hsin Yang, Norain A Talib, Noriaki Ikeda, Tony Axell, Prakash C Gupta, Yujiro Handa, Norlida Abdullah, Rosnah B Zain

**Affiliations:** 1 Oral Cancer Research & Coordinating Centre, Faculty of Dentistry, University of Malaya, Kuala Lumpur, Malaysia; 2 Department of Dental Hygiene, College of Dental Medicine, Kaohsiung Medical University, Taiwan; 3 Oral Health Division, Ministry of Health, Malaysia; 4 Bureau of International Medical Cooperation, National Center for Global Health and Medicine, Tokyo, Japan; 5 Maxillofacial Unit, Halmstad Hospital, Halmstad, Sweden; 6 Healis - Sekhsaria Institute For Public Health, Navi Mumbai, India; 7 Health Sciences University of Hokkaido, Tobetsu (Ishikari), Hokkaido, Japan

## Abstract

**Background:**

Betel quid chewing is a common habit widely practiced in Southern Asian populations. However, variations are seen in the content of a betel quid across the different countries. Factors associated with commencement and cessation of this habit has been numerously studied. Unfortunately, data on Malaysian population is non-existent. This study aims to determine the factors associated with the inception and also cessation of betel quid chewing behaviour among Malaysian adults.

**Method:**

This study is part of a nationwide survey on oral mucosal lesions carried out among 11,697 adults in all fourteen states in Malaysia. The questionnaire included sociodemographic information and details on betel quid chewing habit such as duration, type and frequency. The Kaplan-Meier estimates were calculated and plotted to compare the rates for the commencement and cessation of betel quid chewing behaviour. Cox proportional hazard regression models were used to calculate the hazard rate ratios for factors related to commencement or cessation of this habit.

**Results:**

Of the total subjects, 8.2% were found to be betel quid chewers. This habit was more prevalent among females and, in terms of ethnicity, among the Indians and the Indigenous people of Sabah and Sarawak. Cessation of this habit was more commonly seen among males and the Chinese. Females were found to be significantly more likely to start (p < 0.0001) and less likely to stop the quid chewing habit. Females, those over 40 years old, Indians and a history of smoking was found to significantly increase the likelihood of developing a quid chewing habit (p < 0.0001). However, those who had stopped smoking were found to be significantly more likely to promote stopping the habit (p = 0.0064). Cessation was also more likely to be seen among those who chewed less than 5 quids per day (p < 0.05) and less likely to be seen among those who included areca nut and tobacco in their quid (p < 0.0001).

**Conclusion:**

Factors that influence the development and cessation of this behaviour are gender, age, ethnicity, and also history of smoking habit while frequency and type of quid chewed are important factors for cessation of this habit.

## Background

Areca nut and betel quid with or without tobacco has been declared as Group 1 carcinogens to humans [1]. The role of betel quid chewing habit in the development of oral submucous fibrosis [2] and oral cancer [3] has been substantially studied. Areca nut, which is the main substance in a betel quid, is reported to be one of the most widely used psychoactive substances with several million users around the world, predominantly in Southern Asian countries such as India, Pakistan, Sri Lanka, Papua New Guinea, Taiwan, Mainland China, Myanmar and Thailand [1].

Variations can be seen in the content of a betel quid across the different countries. A typical quid contains areca nut, slaked lime and flavouring ingredients which are wrapped in betel leaf [1] and it is widely practiced in Taiwan, China [4] and Papua New Guinea [5]. Sometimes, tobacco is also added to the quid in some populations such as in India [6], Myanmar and Thailand [7].

In Malaysia, the betel quid chewing habit is prevalent in rural areas among the older generation. In a nationwide survey conducted to obtain baseline data on the prevalence of oral mucosal lesions, the habit of betel quid chewing habit was detected in 7.0% of the Malaysian adults [8], which is comparable to that of the Taiwanese population where it was estimated that the prevalence of the habit was 10% [9].

The factors associated with the commencement and the cessation of this habit has been investigated by several researchers [10-15]. Among the factors cited for the development of this habit are education level, ethnicity, cigarette smoking, alcohol drinking, sign of machoism and social norm. However, so far data on only the prevalence of this habit is available for the Malaysian population. Other important parameters such as the factors that influence the development or stopping of this habit have never been investigated before. Such data would be imperative to provide an insight into the factors that leads to the initiation and cessation of this habit which would be useful in designing intervention programs. Therefore, it is the aim of this study to determine the factors associated with the commencement and also cessation of the betel quid chewing behaviour among Malaysians.

## Methods

### Study population and survey sampling design

This nationwide survey covering the Peninsula Malaysia and East Malaysia (Sabah & Sarawak - parts of Borneo Island) was carried out among adults aged 25 years old and above. The study population from all the fourteen states is made up of all the different ethnic groups in Malaysia. The survey was designed to cover adults living in private households and hence excluded persons residing in institutions such as hostels, hospitals, prisons, boarding houses and military barracks. This survey is part of a major survey carried out to determine the prevalence of oral mucosal lesions in Malaysia. The sample size required for this survey, which was calculated based on the lowest prevalence of major lesions observed in a previous survey (Dental Epidemiological Survey of Adults in Peninsular Malaysia, 1977) [16] was approximately 11,000-12,000. This calculation is based on significance level of 0.05 and at least 80% power of study. A two-stage stratified random sampling was designed to collect study participants. In the first stage, Enumeration Blocks (EB) was selected from the strata within the states. EBs is geographically contiguous areas of land with identifiable boundaries within sub districts or Local Authority areas. All EBs are formed within legally designated gazetted areas with each EB having around 80-120 households. In the second stage, a systematic sample of living quarters (LQ) with a random start was selected from within the EBs where all adults in the selected LQ were examined by the examiners. Ethical approval for this survey was obtained by the Medical Ethics Sub-committee, Research Review Committee, Ministry of Health Malaysia.

### Data collection

Data on the practice of habits and cessation was collected using a standard structured pre-designed questionnaire. Data was collected by recording clerks who were trained to conduct interviews using this instrument. The instrument was a check off type, which was pre-tested prior to the study. The questionnaire included demographic information, practice of risk habits and also the prevalence of oral mucosal lesions. The information on risk habits includes details of the practice of betel quid chewing, alcohol consumption and smoking habit such as age of habit commencement, type, frequency, duration and habit cessation. In this paper, only data on the habit of betel quid chewing will be presented. For the purpose of analysis, a current chewer is defined as a person who is currently chewing or has stopped chewing for less than 6 months, while an ex chewer is a person who has stopped chewing for at least 6 months.

### Statistical analysis

The Kaplan-Meier estimates were calculated and plotted to compare the rates for the commencement and cessation of betel quid chewing behaviour in terms of time (age in years). Two sets of analyses were conducted in this study. First, to identify factors related to the commencement of chewing habit, where all participants were included in the analysis. The event was defined as a person who started betel quid chewing behaviour. The period for the time-to-event was length of time between birth to the age of starting betel quid chewing behaviour. For the event of starting chewing, the follow-up time for censored observation is the age when interviewed by the survey. Second, to identify factors related to the cessation of betel quid chewing, only betel quid chewers were included in the analysis. The event was the person who had stopped chewing behaviour for at least six months. The period for the time-to-event was length of time between age of start chewing behaviour to the age of stop betel quid chewing. For the event of stopping chewing, the follow-up time for censored observation is the years of chewing when interviewed by the survey. The log-rank tests were used to compare the differences among the groups. Furthermore, univariate and multivariate Cox proportional hazard regression models were used to calculate the hazard rate ratios for factors related to commencement or cessation of chewing habits. The proportional hazard assumptions were validated for all of the independent variables in Cox proportional hazard regression models. P-values lower than 0.05 were considered to be statistically significant. These analyses were conducted by the statistical software SAS V9 (SAS Institutes Inc., Cary, NC, USA).

## Results

A total of 11,697 respondents were recruited in this study. The age of the subject ranged between 25 years and 115 years old with a median of 42 years old. There were slightly more female respondents (59.8%) compared to males. This multi ethnic population was made up predominantly by the Malays (55.8%), followed by the Chinese (23.9%), Indians (10%), Indigenous people (9.1%) and others (1.2%) which is in accordance to the population distribution of Malaysia. Of the total subjects, 2,807 (24.0%) were smokers, 633 (5.4%) were consumers of alcoholic beverages and 963 (8.2%) were betel quid chewers.

**Figure 1:**
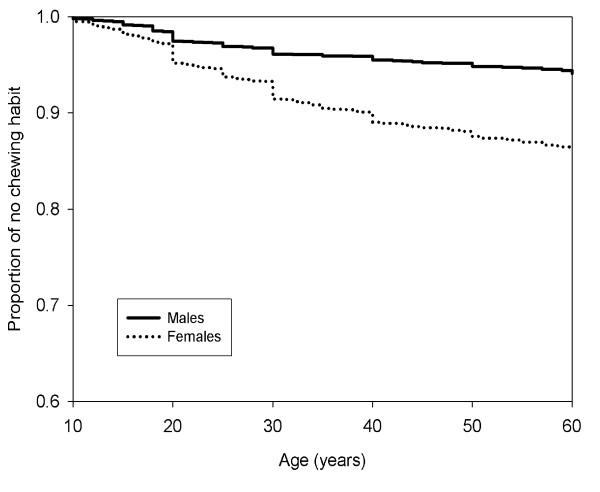
**From birth to commencement of betel quid chewing between males and females**.

The prevalence of the habit of betel quid chewing of Malaysians is shown in Additional file 1. Females had a higher prevalence of the habit (10.5%) as compared to males (4.8%). In each of the variables, females had a higher quid chewing rate than males. This habit was most commonly seen among those above 50 years old for both genders. With regards to ethnicity, Indian females were found to have the highest prevalence (28.9%), followed closely by the Sabah & Sarawak Indigenous females (28.4%). In this population, apart from betel quid chewing, other risk habits such as smoking and alcohol drinking were also prevalent. The practice of multiple habits were seen where among male smokers, 5.8% of them were found to be quid chewers, while among female smokers, 38.8% of them were also chewers. However, it should be noted that based on a population basis of males and females, 3.0% of males are both a smoker and a chewer (142/4698) as compared to 1.9% seen in females (133/6999). Similarly, there is a higher percentage (0.8%) of concurrent drinkers and chewers among males compared to females (0.4%). When considering years of age to start the chewing habit, the Kaplan-Meier plots (Figure 1) showed that both males and females started chewing habit uniformly distributed over the study range of ages.

**Figure 2:**
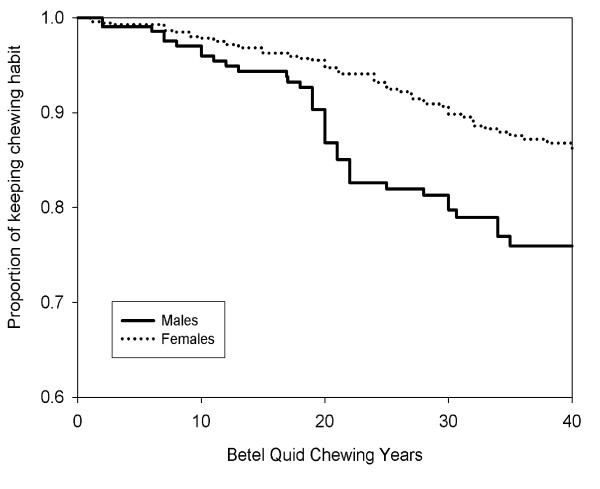
**Commencement to cessation of betel quid chewing between males and females**.

Additional file 2 shows the proportion of chewers who stopped the chewing habit. In general, more males (22.1%) were found to stop quid chewing habit as compared to females (9.6%). The cessation of this habit was significantly more commonly seen among the Chinese for both males and females. Among the females, those who stopped the chewing habit were also drinkers for more than 26 years while among the males, in terms of frequency, those who stopped chewing were drinking less than once a week. Cessation of the habit was most common among those who chewed for 20-29 years and less than 5 quid per day previously. Quitting is also significantly less likely among those who included areca nut in their quid and also practiced tobacco smoking. When considering years of time to stop the chewing habit, Figure 2 showed that the curve for developing the habit for females were above the curve for males, indicating that females were more likely to keep betel quid chewing habit. In addition, one can observe a large decrease in proportion of keeping the habit for males after 15 to 25 years of chewing.

To investigate the factors involved in developing and stopping betel quid chewing habit, the hazard rate ratios (HRR) were estimated using Cox regression analysis. Additional file 3 shows that with regards to developing chewing habits, almost all factors investigated found statistically significant results. Females (HRR = 5.0, 95%CI 4.2-6.0) and those aged more than 40 years (HRR = 2.4 95%CI 1.7-3.2) were found to be more likely to develop the habit. In terms of ethnicity, the Indians had the highest affinity (HRR = 3.9, 95%CI 3.3-4.5), followed by the Sabah & Sarawak Indigenous people (HRR = 3.2, 95%CI 2.7-3.7). Current and past history of smoking was also found to significantly increase the likelihood of developing quid chewing habit. The analysis of factors associated with the cessation of quid chewing habit is shown in Additional file 4. Females (HRR = 0.82, 95%CI 0.5-1.3) and those aged more than 40 years (HRR = 0.2, 95%CI 0.1-0.6) were found to be less likely to stop the habit. Univariate analysis indicated that the Indians were the least likely to stop chewing (HRR = 0.1, 95%CI 0.1-0.3). However, this finding became non-significant in the multivariate model. It was also found that those who have stopped smoking to be significantly more likely to stop the habit (HRR = 2.3, 95%CI 1.3-4.1) but no such association was found for alcohol consumers. The frequency and type of betel quid chewed also plays a role in the cessation of the habit. Cessation was more likely to be seen among those who chew less that 5 quids per day and less likely to be seen among those who included areca nut and tobacco in their quid.

## Discussions

This article is based on information in a nationwide survey that was carried out to determine the prevalence of oral mucosal lesions (OML) in Malaysia. Among the information collected was the prevalence of risk habits for OML such as smoking, betel quid chewing and alcohol consumption. This paper thus focuses on the betel quid chewing habit and explores the possible factors that influence the inception and cessation of this habit.

Usually logistic regressions are used to identify the related factors for commencement and cessation of betel quid chewing habit. A limitation to this approach would be participants' age. A younger participant is less likely to have chewing habit and may develop the habit at a later age. This is why an increasing chewing prevalence rates in older ages was always found. The advantage of survival analysis on censored events can be used to overcome this situation. From the Kaplan-Meier plots, it is possible to find the changes on proportions of chewing habit over years of age, and proportions of keeping chewing habit over the chewing years. Using the recall time duration from a cross sectional survey data could also be a limitation to this study, since recall bias can easily arise.

The habit of betel quid chewing among Malaysians is more prevalent among females (see Additional file 1). This trend is also evident among other South-east Asian populations such as among the Cambodians [17]. However, in most societies, where this habit is still highly practiced, such as in India [18,19], Taiwan [13,20] and Solomon Islands [12] the habit is more commonly practiced among the males compared to females. The trend of concurrently practicing more than one risk habit was also found in this population (see Additional file 1). This finding is in concordance with other studies which found that the practice of betel quid chewing is usually accompanied by the practice of other risky habits such as smoking and alcohol consumption [20-22]. This practice of multiple risk habits was also found to be more common among males, which is in line with other populations that practiced betel quid chewing.

Apart from the difference in gender distribution of this habit, another variation that can clearly be seen in this multiethnic population is the difference in the distribution of this habit among the different ethnic groups (see Additional file 1). For the males, betel quid chewing was predominantly seen among the 'others' group while for the females, it was found to be most commonly practiced among the Indians, which is the third largest ethnic group in Malaysia. Interestingly, based on the report by the Malaysian National Cancer Registry [23] for the year 2003-2005, cancer of the oral cavity is among the top ten types of cancers among the Indians, while it is not evident among the other ethnic groups. The higher prevalence of this habit among Indians is also in concordance to the findings from an earlier epidemiological survey done by Hirayama et al. [24].

It is noted that from this present study, the results showed that there were no obvious years of ages where more participants tend to develop chewing habit (Figure 1). However, it can be observed that there is a large decrease in male current chewers after 15 to 25 years of chewing (Figure 2). Many factors are thought to influence the inception of this behaviour. A study among adolescents in Taiwan cited the reasons for starting the habit as curiosity, keeping warm and peer pressure [25] while another study quoted the initiation of the habit was as a way to pass the time, tension in the family and boredom [26]. This survey found that females were less likely to stop quid chewing habit as compared to males (see Additional file 2) which corroborates an earlier finding on a sample of Taiwanese adults [11]. This could be attributed to the fact that the female chewers in this population are housewives residing in plantations and were from low socioeconomic status where they face boredom, financial or social problems in the family and also peer pressure where betel quid chewing is considered as a traditional cultural norm, thus contributing to the low cessation rate among the females.

In this multiethnic population, cessation of betel quid chewing habit was more significantly seen among the Chinese for both males and females (see Additional file 2). This scenario is most probably due to the fact that this habit is rare among the Chinese, therefore creating a conducive factor providing a more supportive environment for cessation as compared to the other ethnic groups.

This study identified gender, age, ethnicity and smoking history as the factors that influence the development and cessation of this habit. Females were found to be less likely to quit the habit. This finding is in line with the results from a Taiwan study where females were found to have a lower cessation rate than males [11]. Those aged more than 40 years were found to be significantly more likely to start (see Additional file 3) and also less likely to quit the habit (see Additional file 4). This could be attributed to the fact that this habit is more prevalent among those in the older generation. Furthermore, the foul-smelling breath associated with betel quid chewing [18] might be the reason why the younger generation avoids the behaviour. As for smoking history, those with current and past history of smoking were more likely to start chewing habit (see Additional file 3) while ex-smokers were more likely to quit the habit (see Additional file 4). This finding is in concordance with an earlier study among the Taiwanese which found that smokers are 10 times more likely to become a quid chewer while ex-smokers were twice as likely to quit the habit [20].

The cessation of the quid chewing habit is also influenced by the frequency of chewing and the type of quid chewed. Cessation is significantly more likely among those in the lowest category of chewing frequency who chewed less than 5 quids per day (see Additional file 4). This is in concordance with the finding by Lin et al. [13] in Taiwan where those who are chewing smaller amount of quids per day are more likely to quit their habit. Cessation was also less likely for those chewing quids containing areca nut and tobacco (see Additional file 4). This might be attributed to the contents of arecoline in the areca nut which is a para-sympathomimetic agent that stimulates a sense of well-being, heightened alertness and reduction of tension [27] and the nicotine content in tobacco which is an addictive substance that creates dependency, making it difficult for consumers to stop the habit.

## Conclusions

Factors that influence the development and cessation of this behaviour are gender, age, ethnicity, and also history of smoking habit while frequency and type of quid chewed are important factors for cessation. The habit of betel quid chewing, which has been linked to oral submucous fibrosis (OSF) and also oral cancer is believed to be due to the low awareness level on the carcinogenic effect of this behaviour. Therefore, health policies targeted towards an increase in awareness particularly for the high risk groups identified in this study through educational efforts is urgently needed so that the prevalence of potentially malignant and/or malignant lesions due to this risky behaviour could be reduced. Reasons for developing this habit and the factors that drives them to quit should also be further investigated through qualitative research to promote better understanding so that information derived could be incorporated into health promotion programs to encourage those affected to quit.

## Competing interests

The authors declare that they have no competing interests.

## Authors' contributions

WMNG have made substantial contribution in the conception of manuscript framework, interpretation of data analysis and has drafted the manuscript. IAR helped conceived of the study, participated in the study design conception and coordination and helped to critically revise the manuscript. YHY performed the statistical analysis and helped to revise the manuscript. NAT and NA helped conceived and participated in the study design conception and coordination and helped revised the manuscript. NI helped in acquisition of funding and involved in study design conception and coordination. PCG is involved in study design conception and coordination and provided substantial contribution to sampling estimation. TA and YH helped conceived and participated in the study design conception and coordination. RBZ conceived the study design, led and coordinate the study/research group, ensure quality control of data and helped to critically revise the manuscript. All authors read and approved the final manuscript.

## Pre-publication history

The pre-publication history for this paper can be accessed here:

http://www.biomedcentral.com/1471-2458/11/82/prepub

## Supplementary Material

Additional file 1 **Prevalence of betel quid chewing habit between men and women for different demographic characteristics**. Table S1 tabulated the practice of betel quid chewing across different sociodemographic characteristics of the study population such as age, ethnicity, smoking habit and drinking habit. Click here for file

Additional file 2 **Proportion of chewers stopped chewing between men and women in different demographic characteristics**. Table S2 tabulated the cessation of betel quid chewing habit across different sociodemographic characteristics such as age, ethnicity, smoking habit, drinking habit and duration/frequency of betel quid habit. Click here for file

Additional file 3 **Univariate and multivariate analysis of chewing habit from birth until commencement**. Table S3 shows the results of univariate and multivariate analysis of the association between selected variables and commencement of betel quid chewing habit. Click here for file

Additional file 4 **Univariate and multivariate analysis of chewing habit from inception until cessation**. Table S4 shows the results of univariate and multivariate analysis of the association between selected variables and cessation of betel quid chewing habit. Click here for file
