# Supplementary material for: Factors affecting commencement and cessation of betel quid chewing behaviour in Malaysian adults
Source: BMC Public Health. 2011 Feb 7;11:82. doi: 10.1186/1471-2458-11-82 (PMC3039591; doi:10.1186/1471-2458-11-82)
Supplement: Additional file 1 — Prevalence of betel quid chewing habit between men and women for different demographic characteristics. Table S1 tabulated the practice of betel quid chewing across different sociodemographic characteristics of the study population such as age, ethnicity, smoking habit and drinking habit. [file 1471-2458-11-82-S1.PDF]

Table 1. Prevalence of betel quid chewing habit between men and women for different demographic characteristics

Variables	Item	Males (n=4698)			Females (n=6999)		
		Total subjects	No of chewers, n (%)	p value	Total subjects	No of chewers, n (%)	p value
Total		4698	226 (4.8)		6999	737 (10.5)	
Age group	25-30	676	9 (1.3)	<.0001	1297	43 (3.3)	<.0001
	31-40	1237	25 (2.0)		2270	120 (5.3)	
	41-50	1044	48 (4.6)		1522	143 (9.4)	
	51+	1741	144 (8.3)		1910	431 (22.6)	
Ethnicity	Malays	2605	128 (4.9)	<.0001	3924	334 (8.5)	<.0001
	Orang Asli	24	1 (4.2)		24	6 (25.0)	
	Indigenous people	432	35 (8.1)		580	165 (28.4)	
	Chinese	1097	7 (0.6)		1701	13 (0.8)	
	Indians	477	43 (9.0)		689	199 (28.9)	
	Others*	63	12 (19.0)		81	20 (24.7)	
Smoker	Yes	2464	142 (5.8)	0.0014	343	133 (38.8)	<.0001
	No	2234	84 (3.8)		6656	604 (9.1)	
Years smoking	0-19	1165	33 (2.8)	<.0001	125	41 (32.8)	0.1244
	20-29	663	40 (6.0)		85	39 (45.9)	
	30+	1070	102 (9.5)		199	83 (41.7)	
Alcohol drinker	Yes	517	37 (7.2)	0.0082	116	26 (22.4)	<.0001
	No	4181	189 (4.5)		6883	711 (10.3)	

Table 1. Prevalence of betel quid chewing habit between men and women for different demographic characteristics (*cont*)

Variables	Item	Males (n=4698)			Females (n=6999)		
		Total subjects	No of chewers, n (%)	p value	Total subjects	No of chewers, n (%)	p value
Years drinking	0-10	141	8 (5.7)	0.7247	47	11 (23.4)	0.1761
	11-25	209	16 (7.7)		42	6 (14.3)	
	26+	167	13 (7.8)		27	9 (33.3)	
Frequency of drinking/week	<1 time	257	13 (5.1)	0.0597	69	15 (21.7)	0.4612
	1-2 times	110	8 (7.3)		23	6 (26.1)	
	3-5 times	51	8 (15.7)		4	2 (50.0)	
	Almost daily	99	8 (8.1)		20	3 (15.0)	

*Others: All other ethnic groups that does not fall into the stated categories, ie mixed parentage, etc
