# Supplementary material for: Factors affecting commencement and cessation of betel quid chewing behaviour in Malaysian adults
Source: BMC Public Health. 2011 Feb 7;11:82. doi: 10.1186/1471-2458-11-82 (PMC3039591; doi:10.1186/1471-2458-11-82)
Supplement: Additional file 2 — Proportion of chewers stopped chewing between men and women in different demographic characteristics. Table S2 tabulated the cessation of betel quid chewing habit across different sociodemographic characteristics such as age, ethnicity, smoking habit, drinking habit and duration/frequency of betel quid habit. [file 1471-2458-11-82-S2.PDF]

Table 2. Proportion of chewers stopped chewing between men and women in different demographic characteristics

Variables	Item	Males (n=226)			Females (n=737)		
		Total no of chewers	No of stopped chewing, n (%)	p value	Total no of chewers	No of stopped chewing, n (%)	p value
Total		226	50 (22.1)		737	71 (9.6)	
Age group	25-30	9	2 (22.2)	0.7854	43	2 (4.7)	0.0163
	31-40	25	6 (24.0)		120	8 (6.7)	
	41-50	48	8 (16.7)		143	7 (4.9)	
	51+	144	34 (23.6)		431	54 (12.5)	
Ethnicity	Malays	128	41 (32.0)	0.0001	334	43 (12.9)	<.0001
	Orang asli	1	0 (0.0)		6	0 (0.0)	
	Indigenous people	35	3 (8.6)		165	17 (10.3)	
	Chinese	7	3 (42.9)		13	6 (46.2)	
	Indians	43	0 (0.0)		199	5 (2.5)	
	Others*	12	3 (25.0)		20	0 (0.0)	
Smoker	Yes	142	28 (19.7)	0.2573	133	13 (9.8)	0.9515
	No	84	22 (26.2)		604	58 (9.6)	
Years smoking	0-19	33	8 (24.2)	0.9317	41	9 (22.0)	0.6445
	20-29	40	11 (27.5)		39	6 (15.4)	
	30+	102	28 (27.5)		83	13 (15.7)	

Table 2. Proportion of chewers stopped chewing between men and women in different demographic characteristics (*cont*)

Variables	Item	Males (n=226)			Females (n=737)		
		Total no of chewers	No of stopped chewing, n (%)	p value	Total no of chewers	No of stopped chewing, n (%)	p value
Alcohol drinker	Yes	37	5 (13.5)	0.1677	26	3 (11.5)	0.7375
	No	189	45 (23.8)		711	68 (9.6)	
Years drinking	0-10	8	1 (12.5)	0.9704	11	0 (0.0)	0.0406
	11-25	16	2 (12.5)		6	0 (0.0)	
	26+	13	2 (15.4)		9	3 (33.3)	
Frequency of drinking/ week	<1 time	13	5 (38.5)	0.0136	15	2 (13.3)	0.4777
	1-2 times	8	0 (0.0)		6	0 (0.0)	
	3-5 times	8	0 (0.0)		2	0 (0.0)	
	Almost daily	8	0 (0.0)		3	1 (33.3)	
Years chewing	0-19	72	20 (27.8)	0.0227	261	27 (10.3)	0.0475
	20-29	48	15 (31.3)		149	21 (14.1)	
	30+	106	15 (14.2)		327	23 (7.0)	
No of quid chewed/ day	0-4	160	47 (29.4)	0.0002	467	64 (13.7)	<.0001
	5-9	35	2 (5.7)		163	6 (3.7)	
	10+	31	1 (3.2)		107	1 (0.9)	
Areca chewing/ tobacco smoking	Areca + tobacco	50	0 (0.0)	<.0001	280	5 (1.8)	<.0001
	Areca only	149	34 (22.8)		410	42 (10.2)	
	Tobacco only	6	0 (0.0)		12	0 (0.0)	
	No areca + tobacco	21	16 (76.2)		35	24 (68.6)	

* Others: All other ethnic groups that does not fall into the stated categories, ie mixed parentage, etc
