# Supplementary material for: Factors affecting commencement and cessation of betel quid chewing behaviour in Malaysian adults
Source: BMC Public Health. 2011 Feb 7;11:82. doi: 10.1186/1471-2458-11-82 (PMC3039591; doi:10.1186/1471-2458-11-82)
Supplement: Additional file 3 — Univariate and multivariate analysis of chewing habit from birth until commencement. Table S3 shows the results of univariate and multivariate analysis of the association between selected variables and commencement of betel quid chewing habit. [file 1471-2458-11-82-S3.PDF]

Table 3. Univariate and multivariate analysis of chewing habit from birth until commencement

Variables	Items	Univariate			Multivariate		
		Hazard rate ratio	95% CI	p value	Hazard rate ratio	95% CI	p value
Gender	Male	1.00†			1.00†		
	Female	2.39	(2.06, 2.78)	<0.0001	5.00	(4.16, 6.00)	<0.0001
Age	25-30	1.00†			1.00†		
	31-40	1.24	(0.90, 1.72)	0.1891	1.39	(1.00, 1.92)	0.0476
	41-50	1.98	(1.44, 2.71)	<0.0001	2.36	(1.72, 3.23)	<0.0001
	51+	3.87	(2.89, 5.19)	<0.0001	4.55	(3.38, 6.12)	<0.0001
Ethnicity	Malay	1.00†			1.00†		
	Orang Asli	1.94	(0.87, 4.34)	0.1065	2.27	(1.01, 5.08)	0.0462
	Indigenous people	3.23	(2.73, 3.81)	<0.0001	3.15	(2.65, 3.73)	<0.0001
	Chinese	0.09	(0.06, 0.15)	<0.0001	0.09	(0.06, 0.14)	<0.0001
	Indian	3.29	(2.81, 3.84)	<0.0001	3.86	(3.28, 4.54)	<0.0001
	Others*	3.50	(2.45, 5.01)	<0.0001	3.06	(2.13, 4.38)	<0.0001
Smoker	No	1.00†			1.00†		
	Ex	1.47	(1.14, 1.91)	0.0035	2.92	(2.20, 3.88)	<0.0001
	Current	1.24	(1.07, 1.43)	0.0034	2.33	(1.95, 2.79)	<0.0001
Alcohol drinker	No	1.00†			1.00†		
	Yes	1.20	(0.93, 1.54)	0.1689	1.08	(0.82, 1.42)	0.6055

† Reference category

* Others: All other ethnic groups that does not fall into the stated categories, ie mixed parentage, etc
