# Supplementary material for: Factors affecting commencement and cessation of betel quid chewing behaviour in Malaysian adults
Source: BMC Public Health. 2011 Feb 7;11:82. doi: 10.1186/1471-2458-11-82 (PMC3039591; doi:10.1186/1471-2458-11-82)
Supplement: Additional file 4 — Univariate and multivariate analysis of chewing habit from inception until cessation. Table S4 shows the results of univariate and multivariate analysis of the association between selected variables and cessation of betel quid chewing habit. [file 1471-2458-11-82-S4.PDF]

Table 4. Univariate and multivariate analysis of chewing habit from inception until cessation

Variables	Items	Univariate			Multivariate		
		Hazard rate ratio	95% CI	p value	Hazard rate ratio	95% CI	p value
Gender	Male	1.00†			1.00†		
	Female	0.48	(0.33, 0.69)	<0.0001	0.82	(0.51, 1.32)	0.4100
Age	25-30	1.00†			1.00†		
	31-40	0.46	(0.15, 1.43)	0.1775	0.48	(0.14, 1.68)	0.2511
	41-50	0.20	(0.06, 0.63)	0.0061	0.18	(0.05, 0.63)	0.0072
	51+	0.17	(0.06, 0.49)	0.0010	0.11	(0.03, 0.38)	0.0004
Ethnicity	Malay	1.00†			1.00†		
	Orang Asli & Indigenous people[a]	0.65	(0.40, 1.06)	0.0817	1.28	(0.74, 2.21)	0.3726
	Chinese	2.56	(1.28, 5.09)	0.0076	1.69	(0.72, 3.97)	0.2321
	Indian	0.11	(0.05, 0.28)	<0.0001	0.57	(0.20, 1.65)	0.3025
	Others*	0.62	(0.20, 1.97)	0.4175	0.67	(0.19, 2.39)	0.5417
Smoker	No	1.00†			1.00†		
	Ex	6.75	(4.33, 10.52)	<0.0001	2.26	(1.26, 4.05)	0.0064
	Current	1.88	(1.22, 2.87)	0.0039	0.89	(0.53, 1.49)	0.6528
Alcohol drinker	No	1.00†			1.00†		
	Yes	0.99	(0.48, 2.03)	0.9781	0.90	(0.38, 2.17)	0.8200
No of quid chewed/ day	0-4	1.00†			1.00†		
	5-9	0.18	(0.09, 0.37)	<0.0001	0.43	(0.20, 0.90)	0.0247
	10+	0.06	(0.02, 0.25)	<0.0001	0.15	(0.04, 0.63)	0.0091
Type of quid chewed	None	1.00†			1.00†		
	+ areca nut	0.12	(0.08, 0.18)	<0.0001	0.15	(0.06, 0.38)	<0.0001
	+ tobacco	0.06	(0.02, 0.14)	<0.0001	0.12	(0.04, 0.32)	<0.0001
	+ betel leaves	0.16	(0.11, 0.23)	<0.0001	1.20	(0.37, 3.91)	0.7669
	+ lime	0.18	(0.12, 0.26)	<0.0001	0.96	(0.32, 2.87)	0.9374
	+ *gambir*	1.24	(0.86, 1.78)	0.2478	2.19	(1.30, 3.71)	0.0033

† Reference category

[a]Estimates for Orang Asli & Indigenous people are combined due to insufficient sample size (both are minority ethnics)

* Others: All other ethnic groups that does not fall into the stated categories, ie mixed parentage, etc
